# Simplified Sinus Floor Augmentation: An Economical Approach Using a Modified Balloon Technique

**DOI:** 10.7759/cureus.65346

**Published:** 2024-07-25

**Authors:** Mahantesha Sharanappa, Gargi S Deshmukh, Vinay H Vadvadgi

**Affiliations:** 1 Department of Periodontology, Faculty of Dentistry, M. S. Ramaiah University of Applied Sciences, Bangalore, IND; 2 Department of Dentistry, Deshmukh Dental Hospital, Aurangabad, IND; 3 Department of Periodontology, Rural Dental College, Pravara Institute of Medical Sciences, Loni, IND

**Keywords:** alveolar bone atrophy, bone grafting, catheter, dental implant, sinus floor augmentation

## Abstract

Longstanding partial edentulism in the posterior segment of the maxilla is a challenging treatment and typically involves extensive and invasive sinus floor augmentation. Various methods have been employed for sinus floor elevation, including the application of hydraulic pressure using the balloon technique. This case report describes a modified ballooning technique to elevate the sinus floor using a Foley catheter to apply the hydraulic pressure method to elevate the Schneiderian membrane prior to the placement of bone grafts and an endosseous implant in a 37-year-old male patient who presented with an atrophic alveolar ridge height of 3.0 mm in the area of the extracted left first maxillary molar. Sinus floor elevation using the Foley catheter led to a sinus floor elevation of 7 mm and a gain in alveolar bone height of 7.8 mm. The patient was asymptomatic with a stable implant during the one-year follow-up period after prosthetic implant loading.

## Introduction

The extraction of posterior maxillary teeth has been associated with horizontal and vertical alveolar bone loss and underscores the need for additional procedures to improve the quality and quantity of bone [1,2]. To overcome this problem, augmentation of the maxillary sinus floor has emerged as a widely accepted solution to reinforce the maxillary bone in the posterior region [3,4]. However, membrane perforation is the predominant complication encountered in this procedure. The results of various studies indicate that the incidence of this complication ranges from 7% to 60% [5].

Tatum [6] and Boyne and James [7] first described the procedure of direct sinus lift using a lateral approach. However, this procedure is associated with substantial post-operative swelling, discomfort, and complications. In 1994, Summers presented a less invasive technique for indirectly lifting the maxillary sinus floor using a transcrestal approach [8]. However, there is an increased risk of sinus membrane perforation during the course of the procedure in 0-21.4% of cases [9].

The balloon-assisted maxillary sinus floor augmentation (BA-MSFA) technique was developed by Muronoi et al. [10], who adopted a direct approach, and was subsequently modified by Soltan and Smiler [11]. The BA-MSFA procedure has been associated with fewer complications and a gain in alveolar bone height of 4.37-10.47 mm [12]. In 2006, Kfir et al. [13] introduced a technique known as minimally invasive ballooning of the antral membrane, which aims to reduce the incidence of antral membrane perforation and improve the long-term preservation of bone height. However, all these procedures require special instrumentation, which is costly and unaffordable in regions with poor economic conditions. Therefore, we developed an economical method of sinus lift with a readily available armamentarium, which is based on Summers' concept of a direct approach to the maxillary sinus through the lateral window; therefore, it is less invasive and associated with minimal complications. This procedure is presented in the following case report.

## Case presentation

**Figure 1:**
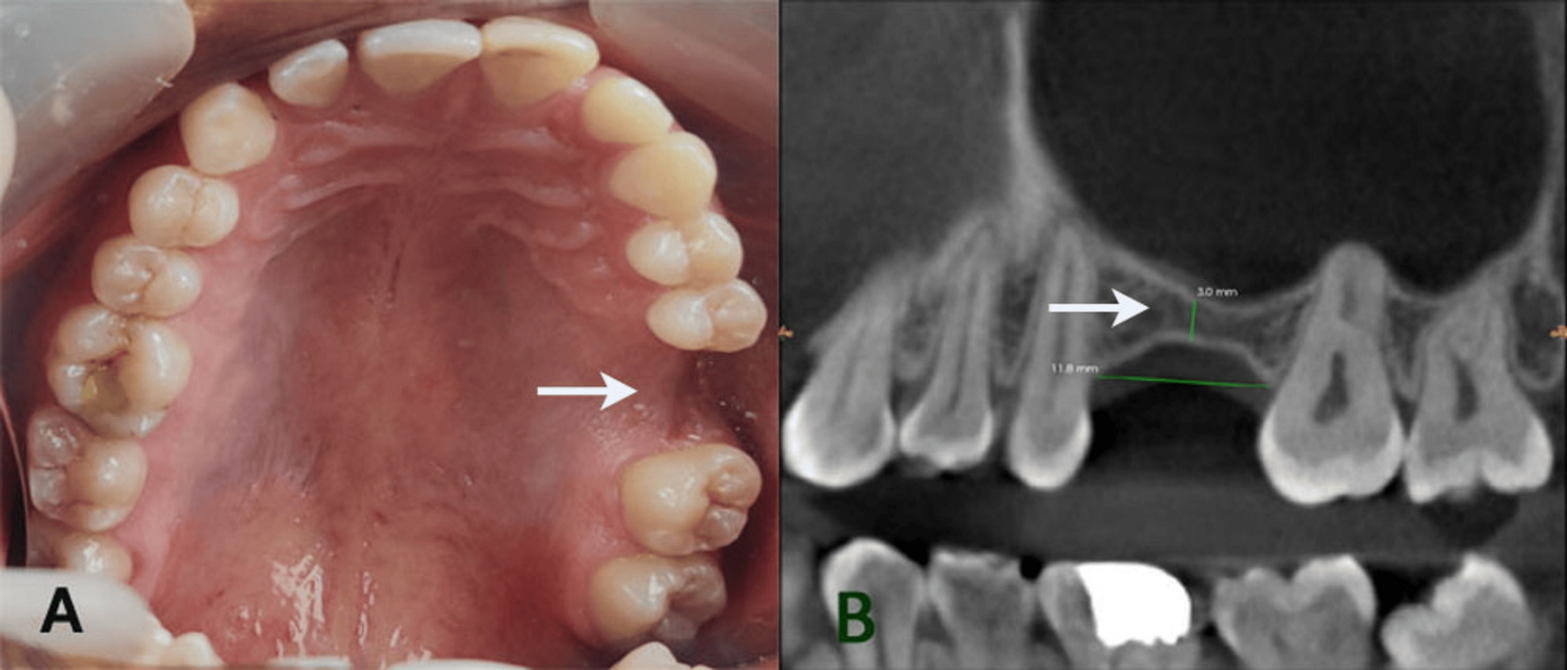
Intraoral view of missing #26 (A) and preoperative CBCT showing the alveolar height of 3 mm and width of 11.8 mm in the area of #26 (B) CBCT: cone beam computed tomography

A 37-year-old partially edentulous male reported to the Department of Prosthodontics and Implantology with the chief complaint of a missing left first maxillary molar (#26) (Figure 1A). The patient had no relevant medical history and was systemically healthy. His dental history revealed that #26 was extracted seven years ago due to caries, leading to an atrophic alveolar ridge in that area. Cone beam computed tomography (CBCT) revealed that the height of the alveolar bone from the alveolar crest to the sinus floor of tooth #26 was 3.0 mm, and the width was 11.8 mm (Figure 1B).

According to Engelke et al., when the alveolar bone height is less than 4 mm, the primary stability of dental implants can be achieved using a bone grafting procedure [14]. After clinical and radiological examinations, the treatment plan involves delayed implant placement after graft placement, which requires elevation of the floor of the sinus as an initial step.

Procedure

The diagnostic casts were fabricated before the procedure. Prophylactic antibiotics were administered preoperatively and continued for seven days post-operatively (amoxicillin-clavulanate 500/125 mg three times a day and 0.12% chlorhexidine rinses three times daily). After block infiltration, a crestal incision was made, and the full-thickness mucoperiosteal flap was elevated from the distal surface of the upper left second premolar to 3 mm distal to the edentulous area of #26, along with vertical releasing incisions to facilitate flap elevation.

**Figure 2:**
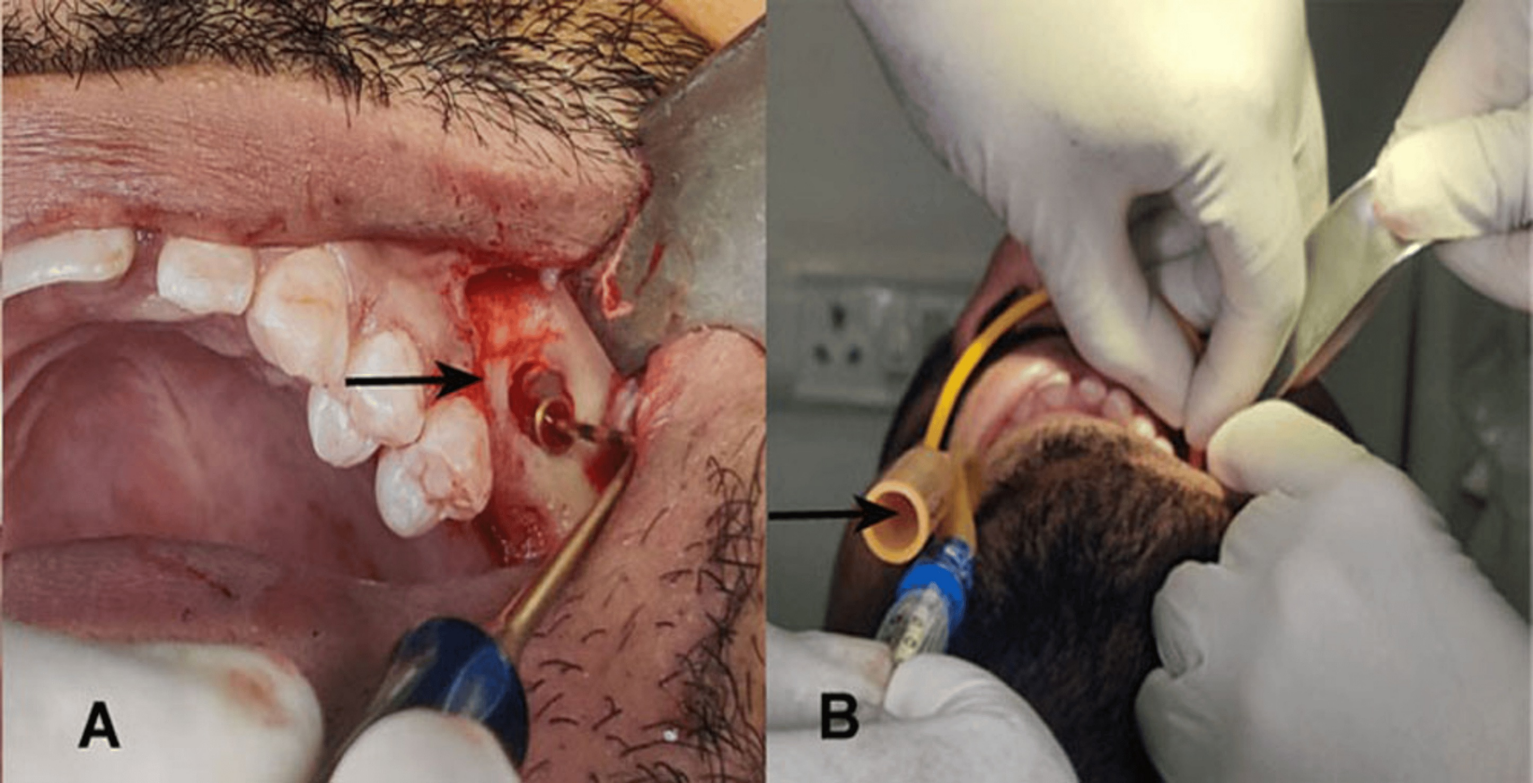
Lateral window approach for maxillary sinus (A) and ballooning technique for sinus lift using No. 6 pediatric Foley catheter (B)

Summer's lateral window method with a direct approach was implemented during the procedure, where a full-thickness mucoperiosteal flap was appropriately reflected and an oval-shaped lateral window was cut using either a high-speed bur or a specific tool (Dentium New Sinus Kit, Dentium, Seoul, Korea). The oval-shaped lateral window was cut with the mesial limit at a minimum distance of 1.5 mm from adjacent teeth. The base of the lateral opening was required to be positioned at least 3 mm from the lower border of the maxillary sinus. Subsequently, the sinus membrane was cautiously reflected and elevated in an inward and upward direction by 7 mm, creating a small space between the sinus membrane and the bony sinus floor, using a surgical curette to avoid accidental perforation (Figure 2A).

Before the Foley catheter balloon was inserted, it was filled with sterile saline solution (2.5 mL) to check for leakage. A pediatric No. 6 Foley catheter (Romsons GS-1090 Uro Cath, CK Medicos, Uttar Pradesh, India) was then attached around the orifice, and pressure was applied (Figure 2B). The relative size of a Foley catheter is described using French units (Fr). In general, urinary catheters range in size from 8 Fr to 36 Fr in diameter. A Fr of 1 is equivalent to 0.33 mm = 0.0113" = 1/77" in diameter. The cross-sectional diameter of a urinary catheter is three times that of the diameter. After the syringe was filled with sterile saline solution and completely extracted from the air, the syringe plunger was slowly depressed. Subsequently, 1.5-2 ml of isotonic saline solution permeated beneath the membrane, leading to its separation from the bony base of the sinus. Consequently, due to hydraulic forces, a lift of the sinus floor was initiated.

The balloon was left in place for five minutes to reduce the elasticity of the membrane, after which it was removed. After removing the Foley catheter, the sinus membrane was checked for perforation using the Valsalva maneuver. This procedure allowed for the placement of the graft material and implants, ranging from 10 to 13 mm in length. A viscoelastic calcium phosphosilicate alloplastic putty graft material (Nova Bone Putty, manufactured by Osteogenics Biomedical) was carefully inserted into the bony defect to reach the submembrane space. Following the bone grafting procedure, a semirigid resorbable collagen barrier membrane (OssMem Hard; Osstem) was applied. The sinus was raised to a level of 7 mm, and the area was sutured with interrupted sutures. In cases where the residual bone height falls below 4 mm, it may be prudent to opt for delayed implant placement [15].

**Figure 3:**
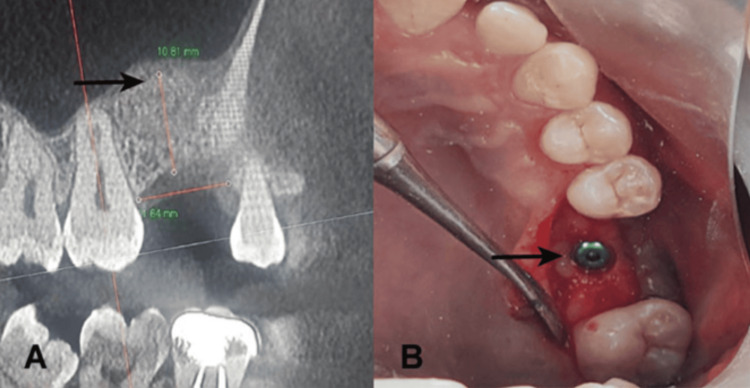
Post-operative CBCT showing an alveolar height of 10.8 mm in the area of #26 (A) and dental implant placement for #26 (B) CBCT: cone beam computed tomography

**Figure 4:**
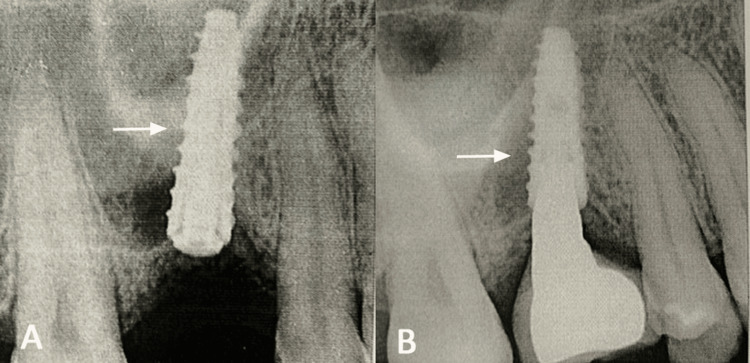
Intraoral periapical radiograph showing the placed dental implant for #26 (A) and at one-year follow-up showing the stable implant (B)

The patient was provided post-operative instructions. Prophylactic antibiotics were administered for five days after the surgical intervention. The patient did not report any post-operative complications during the one-week follow-up visit. Therefore, the patient was recalled for a second surgical procedure after six months. Post-operative CBCT revealed an increase in the alveolar bone height to 10.8 mm (Figure 3A). CBCT confirmed the absence of sinus perforation. The dental implant (4.5 × 10 mm; Osstem, South Korea) was inserted into place using a surgical template (Figure 3b). Intraoral periapical radiography showed successful implant placement (Figure 4A).

The prosthesis was placed after one month, and during the one-year follow-up period, the patient did not report any symptoms of sinus disease, and the implant was also stable as assessed by the intraoral periapical radiograph (Figure 4B). Therefore, according to the implant success criteria of Buser et al., it was 100% [16].

## Discussion

In the present case, Summer’s direct approach with a lateral window was used to assess the maxillary sinus [8], and a hydraulic pressure ballooning technique was used for sinus lift [13]. The lateral window approach is preferable because it allows a more elaborate elevation of the membrane along with its repair [5,9]. The ballooning technique with the lateral sinus approach has been found to be very effective in increasing the alveolar bone height by more than 10 mm with minimal complications [17]. The gentle nature of the technique compared with alternative methods is advantageous for safety. It is more prudent to administer the fluid slowly and gradually, applying only 0.2 ml of fluid at first and then cycling through delicate pushing and aspiration of greater amounts of fluid.

In the present case, the No. 6 pediatric Foley catheter was successfully used to raise the sinus floor. As Foley catheters are available in different sizes, the specific size can be chosen according to the amount of elevation required. At the one-year follow-up after the prosthetic loading of the implant, the implant was stable with no signs of sinus infection or disease. A Foley catheter balloon was effectively used for the frontal sinus by Askar et al. in 2015 [18]. However, to the best of our knowledge, a Foley catheter has not been used to raise the maxillary sinus floor.

An intraoperative risk associated with this technique is the potential inadvertent introduction of fluid into the sinus owing to leakage from the Foley catheter and membrane tears. Both of these issues were addressed in the current case report through a preliminary examination of Foley's catheter balloon for potential leaks and a subsequent inspection of the membrane following lift to identify any perforations using the Valsalva maneuver. This was further corroborated by post-operative CBCT findings. Furthermore, a resorbable barrier membrane was placed, which prevented the extrusion of the graft material into the sinus.

The benefits of the technique described include cost efficiency, the use of easily accessible tools, and reduced invasiveness, which led to a decrease in post-operative discomfort and inflammation, as observed in our patient. Furthermore, it eliminates the need for traditional osteotomes and mallets, which have occasionally been associated with adverse effects on the visual, auditory, and equilibrium functions.

One drawback associated with the use of Foley catheter dilation in elevating the maxillary sinus floor is the potential requirement of a four-handed technique that involves an assistant for balloon inflation. Furthermore, the inability to monitor the pressure within the balloon increases the risk of balloon rupture due to potential overinflation. Therefore, to address this problem, a slow infusion of saline was performed in the present case to prevent overinflation of the balloon and subsequent membrane tears.

## Conclusions

A direct lateral wall approach was implemented in the procedure, which involved sinus floor augmentation utilizing a Foley catheter specifically designed to facilitate the hydraulic elevation of the sinus membrane. This approach also allowed for the precise placement of viscoelastic bone graft material, followed by delayed implant placement. Consequently, this technique yielded a minimally invasive sinus lift balloon surgery, ensuring minimal discomfort for the patient. Moreover, it resulted in a significant increase in alveolar bone height and enhanced dental implant stability. In this case report, a sinus floor elevation of 7 mm and a gain in alveolar bone height of 7.8 mm were observed using a No. 6 Foley catheter. More clinical studies will be required to compare the efficacy of the above-described method.
